# Neural Oscillation Disorder in the Hippocampal CA1 Region of Different Alzheimer's Disease Mice

**DOI:** 10.2174/1567205020666230808122643

**Published:** 2023-10-12

**Authors:** Weiming Yuan, Weijia Zhi, Lizhen Ma, Xiangjun Hu, Qian Wang, Yong Zou, Lifeng Wang

**Affiliations:** 1 Graduate Collaborative Training Base of Academy of Military Medical Sciences, Hengyang Medical School, University of South China, Hengyang, Hunan, 421001, China;; 2 Beijing Institute of Radiation Medicine, 27 Taiping Road, Beijing 100850, China;; 3 Department of Medical Imaging, Chinese PAP Beijing Corps Hospital, Beijing 100600, China

**Keywords:** Alzheimer's disease, hippocampus, neural rhythmic oscillation, local field potentials, theta-gamma phase-amplitude coupling, neurodegenerative disease

## Abstract

**Background:**

Alzheimer's disease (AD) is a well-known neurodegenerative disease that gradually induces neural network dysfunction and progressive memory deficits. Neural network activity is represented by rhythmic oscillations that influence local field potentials (LFPs). However, changes in hippocampal neural rhythmic oscillations in the early stage of AD remain largely unexplored.

**Objective:**

This study investigated neural rhythmic oscillations in 3-month-old APP/PS1 and 5xFAD mice to assess early neural connectivity in AD.

**Methods:**

LFPs were recorded from the hippocampal CA1 region with implanted microelectrode arrays while the mice were in the awake resting stage. Welch fast Fourier transforms, continuous wavelet transforms, and phase-amplitude coupling analyses were used to compute the power density of different frequency bands and phase-amplitude modulation indices in the LFPs.

**Results:**

Our results showed impaired theta, low gamma, and high gamma frequency band power in APP/PS1 and 5xFAD mice during the awake resting stage. AD mice also showed decreased delta, alpha, and beta frequency band power. Impaired theta-low gamma and theta-high gamma phase-amplitude coupling were observed in 5xFAD mice.

**Conclusion:**

This study revealed neural network activity differences in oscillation power and cross-frequency coupling in the early stage of AD, providing a new perspective for developing biomarkers for early AD diagnosis.

## 1 INTRODUCTION

The leading cause of dementia worldwide is Alzheimer's disease (AD), and as the world's population ages, the prevalence of AD has increased [1]. AD is associated with memory deficits and cognitive decline, which are pathologically represented by the formation of nerve plaques, the thickening and tangling of neurofibrils caused by hyperphosphorylation of the intracellular microtubule-associated protein tau, neuroinflammation, apoptosis and neuron loss [2].

Synaptic connective activity is generally thought to change as an index of pathology. One recent study showed that the deposition of Aβ and hyperphosphorylated tau protein is associated with synaptic dysfunction and axonal degeneration in patients with AD [3]. Notably, changes in synaptic activity can also influence cognitive activity of local brain regions through the generation of large-scale rhythmic oscillations [4, 5]. The hippocampus, a deep brain area that produces substantial rhythmic oscillations, is particularly susceptible to damage in the early stages of Alzheimer's disease. Moreover, various intrahippocampal pathological changes affect neural activity dysfunction in patients with AD. Therefore, exploration of hippocampal neural oscillation changes is crucial for diagnosing and preventing the early stage of AD.

The present mechanistic understanding of brain function was derived based on neural signals recorded by extracellular microelectrodes, and precise electrical activity measurements near the electrode tips were used for recording. Local Field Potentials (LFPs) recordings can be applied to assess neural activity, and LFPs can be used to interpret many aspects of neuronal communication with great spatiotemporal resolution [6, 7]. Rhythmic oscillations in neural activity are traditionally classified into canonical frequency bands including: delta (1-4 Hz), theta (4-8 Hz), alpha (8-12 Hz), beta (15-30 Hz), low gamma (30-50 Hz, LG), and high gamma (50-90 Hz, HG) [8]. Analyses of neural rhythmic oscillations may be effective for characterizing the neural mechanisms underlying information communication and neural activity in mesoscopic brains.

Rhythmic oscillation disorders in LFPs have been proposed as functional biomarkers of many neuropsychiatric illnesses [9, 10]. Specifically, patients with AD exhibit uncoupled theta-gamma oscillations, increased delta and theta power, attenuated beta and gamma power, and a general decrease in electroencephalography (EEG) band power [11]. Moreover, the different frequency band oscillations are not isolated phenomena. The amplitudes of high-frequency oscillations can be modified by the phases of low-frequency oscillations, which is termed cross-frequency coupling (CFC) [12]. The CFC between oscillations in neural networks involving the theta phase and gamma amplitude has received considerable attention to date. Many studies have shown that impaired theta-gamma coupling occurs before Aβ accumulation in mouse models of AD, suggesting that alterations in CFC may serve as potential early biomarkers for AD [13, 14].

In the present study, we investigated the changes in network functional connectivity between rhythmic oscillations and their interactions in two mouse models of AD, namely, awake resting-state wild-type APP/PS1 and 5xFAD mice, with *in vivo* electrophysiological experiments. Our results revealed the impaired power of individual frequency bands and the degree of CFC uncoupling, which may be significant for developing early AD biomarkers.

## 2 MATERIALS AND METHODS

### 2.1 Animals

All experiments were performed in accordance with the ethical permit granted by the Institutional Animal Care and Use Committee of the Beijing Institute of Radiation Medicine (IACUC-DWZX-2021-525). Three-month-old healthy male C57BL/6 mice, APP/PS1 mice (Beijing HFK Biotechnology Co., Ltd.), and 5xFAD mice (Shanghai Southern Model Biotechnology Co., Ltd.) weighing 26-30 g were selected. APP/PS1 mice and 5xFAD mice share common characteristics of Aβ deposition, synaptic loss and gliosis at 3 months of age, which is useful in studies on early AD as these characteristics are similar to the preclinical development of AD in humans. Six mice per strain, were used in the experiments, and the mice were housed in breeding cages in a temperature and humidity-controlled room (24°C) under a 12-hour light-dark cycle. Food and water were available ad libitum. Animals were housed in groups before surgery and separately after that. Each procedure was performed in an effort to minimize the suffering of the mice.

### 2.2 *In Vivo* Electrophysiology Surgery and Data Recording

All mice underwent chronic implantation surgery with epidural surface electrodes. Surgery was performed under 1% sodium pentobarbital anesthesia (50 mg/kg, i.p.). The mice were fixed on a digital brain stereolocator after the corneal reflex disappeared, and the horizontal head position was maintained at ± 0.02 mm. The surgical area was sterilized with betadine and 70% ethanol. After exposing the skull, a 1*2 mm rectangular window was cut to implant the microwire array electrode according to the hippocampus stereotactic coordinates (2 mm posterior to the bregma, 1.5 mm lateral to the midline). Then, four screws were driven into the bone near the perimeter, which served as reference ground electrodes and were used for fixation. For the LFPs recordings, a microwire array electrode constructed from 2*4 rectangular array varnish-insulated nichrome wires (35 μm diameter, each with an effective length of 4 mm, electrode spacing of 200 μm, and impedance of 0.5±0.1 Mohm, except for the apical recording point, which was coated with insulating paint) were implanted into the stratum radiatum of the left dorsal hippocampal CA1 area (AP: -2.5 mm, ML: +1.5 mm, DV: -1.5 mm from dura).

**Fig. (1):**
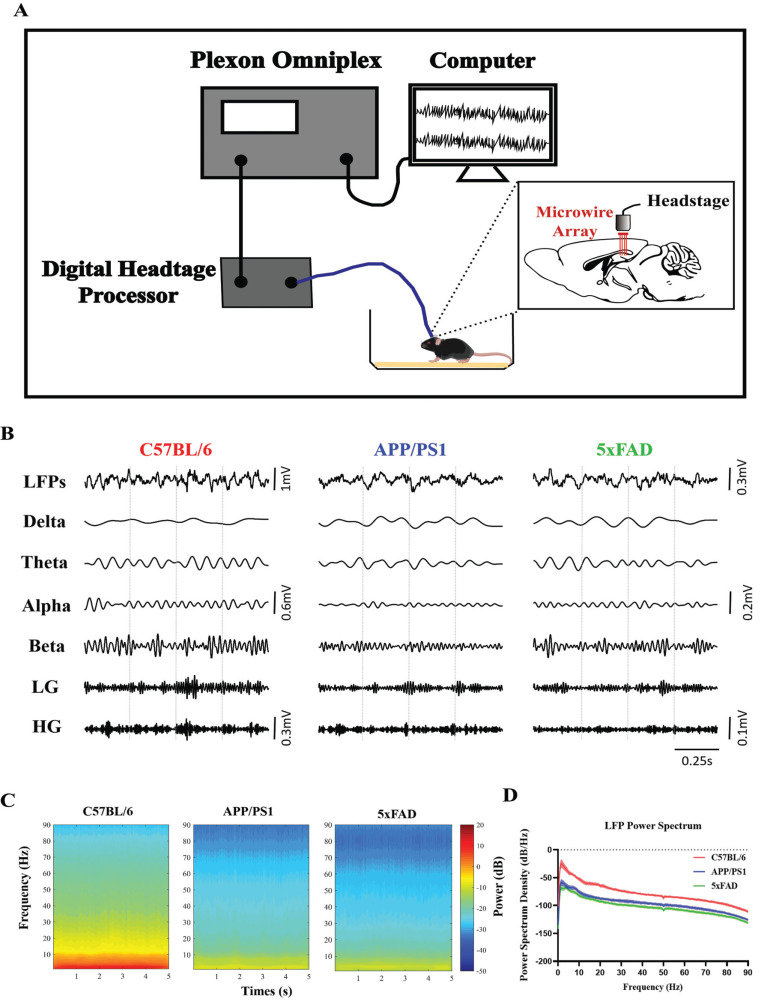
LFPs obtained in the hippocampal CA1 region of AD mice during the awake resting state. (**A**) Diagram of the experimental setup and recording process, which includes implanting microarray electrodes in the mice in the home case as well as, a head-stage front-end amplifier, digital head-stage processor, Plexon Omniplex data acquisition system and computer recording software. (**B**) Examples of raw and filtered recordings from the CA1 region of the hippocampus during the awake resting stage of C57BL/6, APP/PS1 and 5xFAD mice during the awake resting stage. (**C**) Mean time-frequency spectrograms from 1 to 90 Hz in 5 s windows in dB for the C57BL/6, APP/PS1 and 5xFAD groups. The pseudocolor scale indicates the power in dB. (**D**) Mean power spectra from 1 to 90 Hz in dB (C57BL/6: red; APP/PS1: blue; 5xFAD: green). The dashed area indicates the SEM. In (**C**) and (**D**), the power density was transformed to dB with 20*log10.

At 4-5 days after surgery, the mice were placed in their home cage and adapted to the weight of the recording head stage. Then, we continuously recorded electrical signals from the mice during the awake resting stage with a Plexon OmniPlex Neural Recording Data Acquisition System (Plexon Inc., Dallas, TX, USA) and PlexControl software (Fig. **1A**). The raw data were recorded for 5 min at 1000x magnification and digitized with a sampling frequency of 40 kHz. The LFPs data were downsampled at 1 kHz.

### 2.3 Local Field Potential Analysis

The processed LFPs data were imported into MATLAB 2016b using built-in and custom scripts. The recorded LFPs data were first filtered by a 50 Hz notch filter (bandwidth of 3, bandwidth attenuation of 0.04). Then, baseline drifts were removed with a polynomial approach, and the data were bandpass filtered between 1 and 90 Hz using a 2nd infinite impulse response digital filter.

The power spectrum was computed using a fast Fourier transform (FFT) with the Welch method (Hamming windows with lengths of 2 s and 50% overlap) and transformed into decibels (dB) with 20*log10 to determine the power density. For the time-frequency spectrogram, the 5 min LFPs data were divided into 5 s intervals and analyzed via continuous wavelet transforms (CWTs). The transformed power was represented in dB, similar to the FFT process. The delta, theta, beta, alpha, LG and HG band powers were defined as the average power in the frequency ranges of 1-4 Hz, 4-8 Hz, 8-12 Hz, 15-30 Hz, and 30-90 Hz, respectively.

The theta-gamma phase-amplitude coupling (PAC) was measured using a modulation index (MI), as reported in a previous reference [15]. The PAC was computed as follows: First, every 20° interval (0-360°) in the theta rhythm oscillation phase was divided into 18 windows, and the mean amplitudes of the high and low gamma oscillations in each phase bin were computed. In addition, the average amplitude of each phase was normalized by dividing the average amplitude in each phase bin by the total amplitude in each phase bin, resulting in a probability distribution-like function. Finally, the deviation between the phase-amplitude distribution and the uniform distribution was determined using the Kullback‒Leibler (KL) distance, which is frequently used to infer the distance between two distributions [16]. The KL distance was divided by a constant factor (log (number of windows), or the logarithm of the number of phase bins), to ensure that the value was between 0 and 1. This final result of this process is the MI, which varied between 0 and 1. The closer the MI value is to 0, the closer the phase-amplitude distribution is to the uniform distribution and the less relevant the phase-amplitude coupling.

The modulation diagram shown in Figure was obtained by determining the MI values of several narrow-filtered frequency pairs (phase frequencies: 2 Hz bandwidths and 1 Hz steps; amplitude frequencies: 10 Hz bandwidths and 5 Hz steps) and expressing the results with a pseudocolor scheme (see also ref. Tort *et al.* 2009). For the other figures, the MI was calculated between the 4-8 Hz (theta phase), 30-50 Hz (LG amplitude) and 50-90 Hz (HG amplitude) frequency bands. The final LFPs analysis results were obtained by averaging the signals of the 8 channels for each mouse.

### 2.4 Statistical Analysis

All statistical analyses were performed using SPSS 25.0 software and are presented as the mean ± standard error (SEM) by using GraphPad Prism 9.0 software. One-way ANOVA was used to evaluate the rhythmic oscillation power measures after the chi-square test, and the normality test and Tamhane T2 test were applied to compare groups with different variances. The Wilcoxon rank sum test (one-tailed) was used to analyze the MI values. Statistical significance was set at *P <*0.05 and is indicated by * in the figures.

## 3 RESULTS

### 3.1 Oscillation Power Alterations in the LFPs of APP/PS1 and 5xFAD Mice

The hippocampus is crucial for spatial memory formation. Many previous studies on AD mice have shown impaired theta and gamma band power and PAC in the hippocampus and poor performance in behavioral experiments, which suggests that AD mice may have altered hippocampal network activity. To clarify the network changes in different AD model mice, we obtained LFPs recordings in the dorsal hippocampal CA1 region of the mice during the awake resting stage. The LFPs spectrogram was computed in a 5 s window with 1 Hz resolution (Fig. **1C**), and the spectrum was estimated over 5 min (Fig. **1D**). The APP/PS1 and 5xFAD mice exhibited lower power in the LFPs spectrum and spectrogram during the awake resting stage than C57BL/6 mice (Figs. **1B**-**D**).

**Fig. (2):**
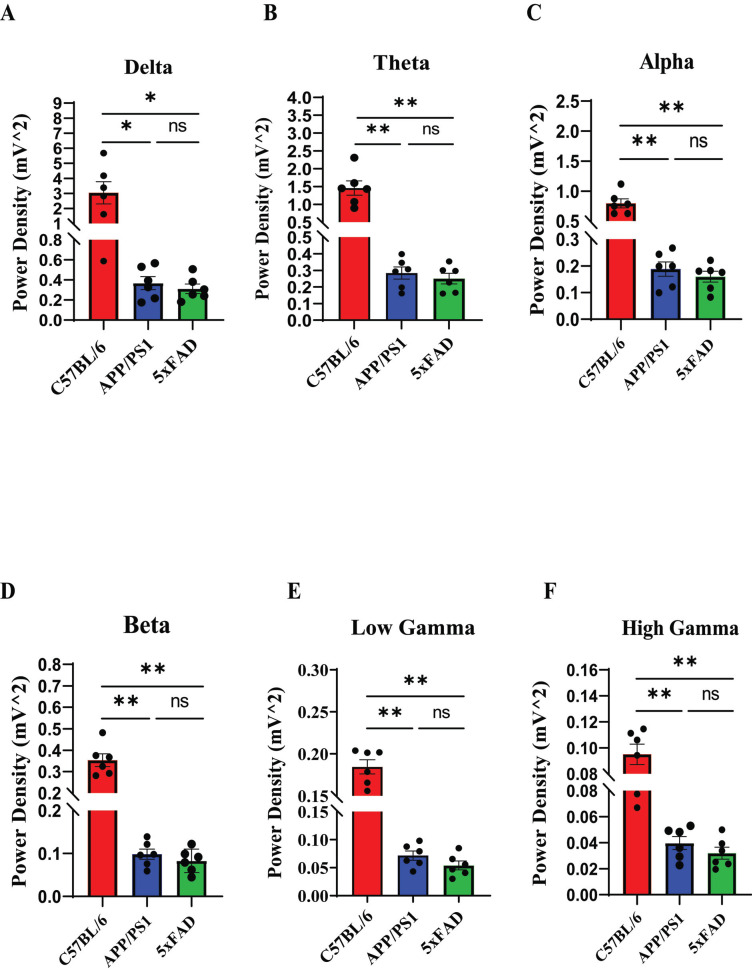
Impaired power of different frequency bands in LFPs from the hippocampal CA1 region of AD mice during the awake resting stage. (**A-F**) Mean power density in different frequency bands in the spectrogram map shown in the bar chart: delta (**A**), theta (**B**), alpha (**C**), beta (**D**), LG (**E**), and HG (**F**). The error bars indicate the SEM. The average power values of different frequency bands were significantly decreased in the APP/PS1 and 5xFAD groups (ns >0.05, *P < 0.05, **P < 0.01).

The average power changes in different frequency bands of the multichannel LFPs are shown in Fig. (**2**). The APP/PS1 group showed considerably decreased power in the theta, LG and HG frequency bands (Figs. **2B**, **E** and **F**). Moreover, the APP/PS1 mice showed impaired delta, alpha, and beta frequency band power (Figs. **2A**, **C** and **D**). Similar results were observed in 5xFAD mice (Figs. **2A**-**F**). Although the average power of the mice in the 5xFAD group was lower than that of the mice in the APP/PS1 group during the awake resting stage, no significant change in any frequency band power was observed (Fig. **2**). These results suggest that the network rhythmic oscillations in the hippocampal CA1 area of APP/PS1 and 5xFAD mice in the awake resting stage are impaired in the early disease progression.

## 3.2 Theta-gamm Coupling in the Different AD Mice

**Fig. (3):**
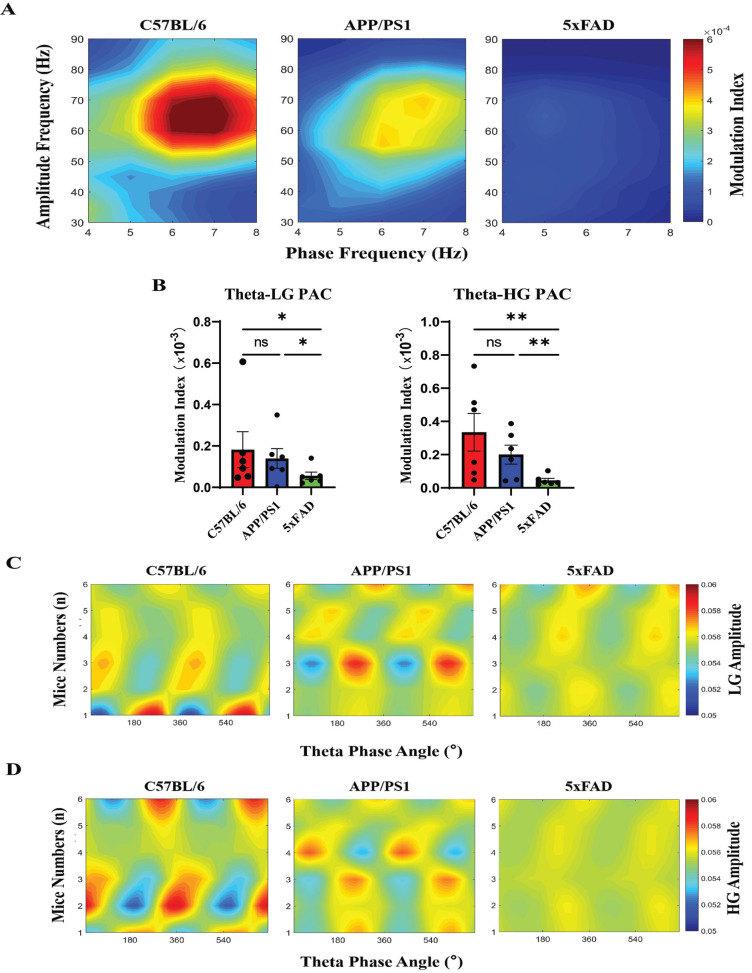
Theta phase modulates the LG and HG amplitude in the hippocampal CA1 area of AD mice during the awake resting stage. (**A**) Mean phase-amplitude modulation diagram of the hippocampal CA1 region of C57BL/6, APP/PS1 and 5xFAD mice obtained during the awake resting stage. The MI is shown by the pseudocolor scale. (**B**) Mean MI strength of theta phase-LG amplitude and theta phase-HG amplitude coupling in the C57BL/6, APP/PS1, and 5xFAD groups (ns P > 0.05, *P < 0.05, **P < 0.01). (**C, D**) LG amplitude (**C**) and HG amplitude (**D**) per theta phase in the pseudocolor maps of each mouse in the C57BL/6, APP/PS1 and 5xFAD groups.

The amplitudes of high-frequency rhythmic oscillations can be modulated by the phases of low-frequency rhythmic oscillations in LFPs. To investigate whether abnormal phase-amplitude coupling occurs in the early stage of AD, we investigated theta-LG and theta-HG coupling in APP/PS1 and 5xFAD mice in the awake resting stage. The extent to which the theta phase modulated the LG and HG frequency amplitudes was assessed by the MI (see Materials and Methods). Furthermore, the theta phase of the associated group, which ranges from 0 to 720°, can reflect the normalized power amplitude per 20° theta phase angle, as shown in the pseudocolor map (Figs. **3C** and **D**). The modulation diagram showed stronger theta-gamma coupling during awake resting stage in the C57BL/6 and APP/PS1 groups than in the 5xFAD group (Fig. **3A**).

Interestingly, when the mean MI of different phase and amplitude ranges (theta phase: 4 to 8 Hz; LG amplitude: 30 to 50 Hz; HG amplitude: 50 to 90 Hz) was used to assess PAC, we found that 5xFAD mice showed significantly weaker theta-LG coupling than C57BL/6 and APP/PS1 mice during the awake resting stage (Fig. **3B**; *p <* 0.05). Theta-HG coupling was also significantly reduced (Fig. **3B**; *p <* 0.01). However, compared with the C57BL/6 mice, the theta phases-LG amplitude and theta phase-HG amplitude coupling were not significantly changed in the hippocampal CA1 area of the APP/PS1 mice during the awake resting stage (Fig. **3B**; *p >* 0.05), although the theta, LG and HG power in the LFPs were impaired (Figs. **2E** and **F**). Taken together, our results suggest that the impaired neural rhythmic oscillations in the hippocampal CA1 area of 5xFAD mice are accompanied by theta-LG and theta-HG uncoupling.

## 4 DISCUSSION

Neural oscillations play crucial roles in neural communication and various memory processes. Neural oscillations can be detected by implanted electrodes in the hippocampus, cortex, striatum, olfactory bulb, thalamus, other brain regions. Although many MEG, EEG, and fMRI studies have indicated that large-scale networks between various brain region are disconnected in patients with AD, a better understanding of LFPs changes in deep brain regions in the early stage of AD is needed. Significant Aβ accumulation, decreased synaptic markers and significantly increased p25 have been reported in 3-month-old 5xFAD mice before cognitive impairment occurs. P25 is generated by Cyclin-dependent kinase 5, which is correlated with synaptic degeneration and neuron loss in 5xFAD mice. Increased p25 may be associated with intraneuronal Aβ aggregates. Moreover, plaques are spread throughout the hippocampus until six months, while the amount of plaque at 10 months of age [17]. Astrogliosis and microgliosis develop in parallel with plaque deposition, which suggests that neuroinflammation occurs early in the 5xFAD model [18]. In contrast, while APP/PS1 mice show reduced APP lyase and Aβ hydrolase, activated microglia, increased astrogliosis, and dendritic spine loss, these mice show no significant Aβ or tau protein accumulation at 3 months of age [19]. The results of *in vitro* and *in vivo* experiments indicate that Aβ deposition and reduced APP can damage gamma frequency band power and theta-LG and theta-HG phase-amplitude coupling in LFPs [20, 21]. However, neural network activity in AD brains has not been well defined. Therefore, in this study, we investigated the neural network activity in the hippocampal CA1 area in 3-month-old APP/PS1 and 5xFAD mice. We evaluated the rhythmic oscillations and coupling in the LFPs recordings, to explore synaptic networks and neural communication in early AD models. Interneuron networks are believed to contribute to the production of theta and gamma oscillations [22, 23]. In general, theta oscillations are related to memory formation, and their disruption impairs spatial memory, while the reversal of theta rhythm oscillations promotes cognitive performance [24]. Gamma oscillations, which are commonly observed in the hippocampus, cortex, striatum, olfactory bulb, thalamus, and other brain regions, promote various cognitive functions, such as attention, perception, object recognition, and memory processes [25, 26]. We found that theta, LG and HG power were impaired in the early stages of both AD mice during the awake resting stage. This result contradicts a previous study, which suggested that LG power was increased in the hippocampal CA1 region in 14-week-old male APP/PS1 mice during the light cycle, although the theta band power results were consistent [27]. Furthermore, HG power in the hippocampal CA1 region of 5xFAD mice decreased under deep anesthesia, and HG power in the DG region of the hippocampus was also decreased [28]. Interestingly, gamma band modulation of noninvasive proof of 40 Hz light and sound can reverse cognitive impairment, attenuate Aβ deposition and recruit microglia [29, 30].

Delta, beta, and alpha oscillations in LFPs are associated with several aspects of cognition. For instance, cognitive functions such as attention are reflected by delta oscillations, which occur by focusing on one stimulus while ignoring other stimuli and during memory consolidation in non-REM sleep periods [31, 32]. In addition, beta oscillations are involved in encoding position information. Furthermore, mice with higher beta oscillations in the CA1 region of the hippocampus perform better in novel object recognition tasks [33]. Moreover, cognitive functions such as memory processing and maintenance require the participation of the alpha band, which suppresses task-irrelevant brain regions and transmits information to task-relevant regions [34]. In our study, we also discovered impaired delta, beta, and alpha frequency band power in the hippocampal CA1 region in APP/PS1 and 5xFAD mice during the awake resting stage. However, decreased alpha, beta, and gamma oscillations in posterior regions and increased delta and theta oscillations have been commonly observed in the EEG data of AD patients during the resting stage [8]. Nevertheless, the discrepancy in the delta and theta oscillations between our results and AD patient EEG data can be attributed to the limitation of EEG in inaccurately reflecting neural rhythmic activity in deep brain regions. In summary, the disruption of different rhythmic oscillations in the LFPs is representative of neural network connectivity disorders in the hippocampal CA1 region of APP/PS1 and 5xFAD mice during the awake resting stage, which may be related to neuronal signaling deficits caused by pathological damage in the early stage of AD.

Theta and gamma oscillations interact with each other, which is known as cross-frequency phase-amplitude coupling. This low-frequency phase-modulated high-frequency amplitude coupling has been observed in particular brain regions during routine cognitive activities [35, 36]. Theta-gamma phase-amplitude coupling can also be characterized by network activity in hippocampal recordings. Previous studies have reported decreased theta-HG coupling in the hippocampal CA1 region of 4-month-old APP-deficient mice during the resting state [20] and 9-month-old mice during the active wakefulness state [37]. Moreover, theta-HG coupling is impaired in the hippocampal region under mild anesthesia in 8- to 9-month-old APP/PS1 mice [14]. Although the results of previous study show that anesthesia application leads to low firing rates in the hippocampus, it remains unclear how theta-gamma coupling is altered in rodents under different states [38]. In contrast, our results showed that theta-LG and theta-HG phase-amplitude coupling were impaired in 5xFAD mice but not in 3- month-old APP/PS1 mice during the awake resting state. Theta-gamma coupling depends on functional synaptic plasticity within functional local and long-range networks, which could be an index of specific network activity [39], although the exact functional significance of theta-gamma PAC remains unclear in the early pathogenesis of AD. Many evidences support the idea that neurological toxicity and damaging effects are determined by the levels of soluble Aβ and plaque accumulation [40-42]. Otherwise, hyperphosphorylated tau protein, synaptic loss, neuron loss, and neuroinflammation can also disrupt neural network connectivity [43-45]. Thus, AD- like pathology disrupts the generation of normal neural rhythmic oscillations and phase-amplitude CFC in the brain. One previous study showed that impaired theta-gamma PAC is occurs in patients with AD-related dementia and mild cognitive impairment (MCI), which worsens with disease progression [36]. In addition, the decline in theta-gamma PAC is the basis of the decline in associative memory in normal cognitive aging [46]. Many previous studies have suggested that Aβ amyloid markers are formed earlier than tau proteins in humans during the preclinical development of AD [47, 48]. The APP/PS1 and 5xFAD mice used in this study have been widely used by many researchers to study the role of Aβ in the development of AD. In these studies, the genetic background of the mice was mainly characterized based on amyloid-related phenotypes as their genetic background. Moreover, the results, suggest that Aβ accumulation, neuroinflammation, and synaptic loss in the hippocampus of 3-month-old 5xFAD mice was more severe than those in APP/PS1 mice at the same age. This may be a possible mechanism for the synchronized impairment of theta-low gamma and theta-high gamma PAC during early the stages of AD.

Our results might indicate that theta-gamma phase-amplitude uncoupling does not occur in the initial stages of Aβ accumulation during the development of AD has not yet occurred. Therefore, theta-gamma PAC disorders in various AD model under different states need to must be studied further.

## CONCLUSION

In conclusion, our results indicate that different frequency band powers are impaired in LFPs oscillation recordings from the hippocampal CA1 areas of 3-month-old APP/PS1 and 5xFAD mice during the awake resting stage. Moreover, impaired theta-LG and theta-HG phase-amplitude coupling were observed in 5xFAD mice. Our results demonstrated neural network activity differences in terms of the oscillation power and cross-frequency coupling in the early stages of AD, providing data support for the development of AD treatments involving neural rhythmic oscillation modulation.

## AUTHORS’ CONTRIBUTIONS

Lifeng Wang and Yong Zou contributed to the study conception and design. Weiming Yuan performed experiments and wrote the draft. Data collection and analysis were performed by Wei Ming Yuan. Xiangjun Hu, Weijia Zhi, Lizhen Ma and Qian Wang discussed the results. All authors read and approved the final manuscript.

## Data Availability

Data and codes during the current study are available from the corresponding author upon reasonable request.

## LIST OF ABBREVIATIONS

AD: Alzheimer´s Disease
LFPs: Local Feild Potentials
CFC: Cross-frequency Coupling
PAC: Phase-amplitude Coupling
LG: Low Gamma
HG: High Gamma
FFT: Fast Fourier Transform
CWTs: Continuous Wavelet Transforms
EEG: Electroencephalography
